# Prevalence, Types, Risk Factors and Clinical Correlates of Anaemia in Older People in a Rural Ugandan Population

**DOI:** 10.1371/journal.pone.0078394

**Published:** 2013-10-23

**Authors:** Joseph O. Mugisha, Kathy Baisley, Gershim Asiki, Janet Seeley, Hannah Kuper

**Affiliations:** 1 MRC/UVRI Uganda Research Unit on AIDS, Entebbe, Uganda; 2 London School of Hygiene and Tropical Medicine, London, United Kingdom; 3 School of International Development, University of East Anglia, Norwich, United Kingdom; Johns Hopkins University, United States of America

## Abstract

**Background:**

Studies conducted in high income countries have shown that anaemia is a common medical condition among older people, but such data are scarce in Africa. The objectives of this study were to estimate the prevalence, types, risk factors and clinical correlates of anaemia in older people.

**Methods:**

Participants were aged (≥ 50) years recruited from a general population cohort from January 2012 to January 2013. Blood samples were collected for assessing hemoglobin, serum ferritin, serum vitamin B12, serum folate, C-reactive protein, malaria infection and stool samples for assessment of hookworm infection. HIV status was assessed using an algorithm for HIV rapid testing. Questionnaires were used to collect data on sociodemographic characteristics and other risk factors for anaemia.

**Results:**

In total, 1449 people participated (response rate 72.3%). The overall prevalence of anaemia was 20.3 % (95% CI 18.2-22.3%), and this was higher for males (24.1%, 95% CI=20.7-27.7%) than females (17.5%, 95% CI=15.0-20.1%). In males, the prevalence of anaemia increased rapidly with age almost doubling between 50 and 65 years (p-trend<0.001). Unexplained anaemia was responsible for more than half of all cases (59.7%). Anaemia was independently associated with infections including malaria (OR 3.49, 95% CI 1.78-6.82), HIV (OR 2.17, 1.32-3.57) heavy hookworm infection (OR 3.45, 1.73-6.91), low fruit consumption (OR 1.55, 1.05-2.29) and being unmarried (OR 1.37 , 95% CI 1.01-1.89). However, the odds of anaemia were lower among older people with elevated blood pressure (OR 0.47, 95% CI 0.29-0.77).

**Conclusion:**

Anaemia control programmes in Uganda should target older people and should include interventions to treat and control hookworms and educational programs on diets that enhance iron absorption. Clinicians should consider screening older people with HIV or malaria for anaemia. Further studies should be done on unexplained anaemia and serum ferritin levels that predict iron deficiency anaemia in older people.

## Introduction

Anaemia is the most common disorder of the blood, and is characterised by a decrease in number of red blood cells or less than the normal quantity of haemoglobin (Hb) in the blood. The haemoglobin value below which anaemia is defined varies, although the World Health Organisation (WHO) Hb thresholds of less than 130 g/L for men and less than 120 g/L for women [1] are the most common definitions used for anaemia even in older people.

 Estimates from WHO show that anaemia affects 1.62 billion people (1.50-1.74) globally and suggest that 23.9% (164 million) of people (≥ 60) years were affected by anaemia [2]. However, the WHO global estimates of anaemia in the older people did not include data from the African region due to lack of survey data from the region. A further systematic review of 45 studies on prevalence of anaemia in older people aged 65 years showed that the overall prevalence of anaemia among older people in high income countries was 17% (3-50%) [3], but did not include any study from the African region. 

A literature search identified only two studies on anaemia in older people conducted in the African region. One study conducted in a peri-urban population of older people (≥65) in South Africa estimated the prevalence of anaemia as 12.5% in males and 13.2% in females [4], while another carried out in a general population of older people (≥65) in Zimbabwe estimated the prevalence of anaemia as 23% [5]. These two African studies are old and their results cannot be generalised to other older people in Sub Saharan Africa. Anaemia studies conducted in high income countries that have included Blacks in their study population show that the prevalence of anaemia is higher among older Blacks compared to older Whites [6]These differences in race have not been clearly explained but some studies suggest genetic differences between Blacks and Whites [7,8].

It is estimated that approximately 50% of anaemia cases worldwide are due to iron deficiency [2], where anaemia occurs as a result of inadequate supply of iron for Hb synthesis. The commonest causes of anaemia in older people in high income countries include nutritional deficiencies, chronic inflammation, chronic kidney disease and anaemia of unknown causes [9]. The causes of anaemia among older people are likely to be different in Africa, and may include soil transmitted helminths, especially hookworm infection, chronic infections such as HIV, malaria infection and nutritional deficiencies [10]. Older people in Africa may be more vulnerable to iron deficiency and Vitamin B12 deficiency which are mainly derived from animal products which older people may not afford. 

To control anaemia, WHO recommends that the magnitude of anaemia together with the contributing factors in the affected group be established and addressed [2]. Despite the fact that the population of older people is increasing in Africa [11] there are scarce data on the epidemiology of anaemia among the older people . These epidemiological data are necessary to inform policy and practice in planning interventions for controlling anaemia in older people.

The General Population Cohort (GPC) of Medical Research Council (MRC)/Uganda Virus Research Institute (UVRI) Uganda Research Unit on AIDS, in Southern Uganda includes a large number of older people and thus provides us an opportunity to obtain an adequate sample size to precisely estimate the prevalence of anaemia, type the anaemia, to study the risk factors and describe the clinical correlates of anaemia among the older people.

## Materials and Methods

### Ethics Statement

The study obtained Ethics approval from the scientific and ethics review boards from London School of Hygiene and Tropical Medicine, Uganda Virus Research Institute and The Uganda National Council for Science and Technology. We obtained written informed consent from all the study participants and all participants requiring medical treatment were referred to the GPC study clinic and nearby government health centres where treatment was offered free of charge. 

### Study setting

The GPC has been described in detail elsewhere [12]. Briefly, the GPC is a population-based cohort study including the residents of 25 villages within the Kyamulibwa sub-county in Kalungu district in rural south-west Uganda. The GPC was established in 1989 by the MRC/UVRI Uganda Research Unit on AIDS to describe trends in the prevalence and incidence of HIV infection and their determinants in the general population. The initial recruitment was conducted in 1989/1990.

Every year since 1989, annual door-to-door “rounds” of census and survey have been carried until 2012 when the door to door survey method was changed and participants were invited and surveyed at central hubs in each of the study villages (a building was rented for the purpose in each village).

During the survey, demographic, socio-medical and serological data are collected from all residents in the study villages. Information regularly obtained includes data on fertility, mortality, migration, sexual behaviour, perceptions of HIV infection and HIV status. In addition to conducting HIV sero surveys, the GPC also provides a population for recruitment for other studies.

The GPC is run from the MRC/UVRI Uganda Research Unit on AIDS field station located in Kyamulibwa Sub County. The GPC runs a clinic and a clinical laboratory at the field office where GPC participants are referred for free treatment if found sick during the survey. The GPC clinic is run by three full time doctors and one clinical officer and is stocked with most of the drugs on the essential drug list for Uganda. Some of the laboratory tests that cannot be performed at the GPC clinical laboratory are performed at the main laboratory located at the head office of MRC/UVRI Uganda Research Unit located in Entebbe.

### Study design

The cross sectional survey for anaemia among the older people was nested within the GPC survey of the MRC/UVRI Uganda Research Unit on AIDS, from January 2012 to January 2013.

### Study population and recruitment

All the people within the GPC who were aged 50 years and above were eligible for inclusion. All older people who were found too ill to undergo the interview and physical measurements and had no other person in the household to help them complete the questionnaire were excluded. Older people within the GPC were first enumerated by a trained census team in their homes, comprising of a team leader and four other members. During the census, baseline socioeconomic and demographic data were collected to provide information which was used to plan for data collection during the survey. After the census, all older people that met the eligibility criteria were asked either to come to the village central hubs or to go to the Kyamulibwa field clinic where data collection and drawing of the study bloods were conducted. 

### Data collection

Data were collected by 5 trained interviewers (2 nurses and 3 none medical trained interviewers) using a questionnaire programmed on ultra mobile personal computers (UMPCs). The questionnaire for the anaemia survey was developed using some existing questions in the GPC survey questionnaire, Study on Global Ageing and adult health (SAGE) [13] questionnaire and Cross Cultural Assessment Of Nutrition in Older Subjects (CRONOS) [14] questionnaire. Questions on co morbidities were added by one of the authors (JM) based on a review from previous studies on anaemia. The questionnaire was translated into the local language and back translated into English by two groups of GPC translators, these versions were compared and necessary adjustments made. The questionnaire was pilot tested on 63 older people. The questionnaire specifically included items on socio demographic characteristics of study participants, tobacco and alcohol use, past history and treatment of some medical conditions (hypertension, stroke, diabetes mellitus, arthritis, intestinal worms, peptic ulcers tuberculosis, cancer, malaria and gastro intestinal bleeding). The questionnaire also contained items on diet and nutritional practices and current use of AZT for participants who were HIV positive and on treatment with antiretroviral drugs.

 For those that were enumerated during the census but did not come to the central hubs or study clinic for data collection, an interviewer visited their homes to establish the reasons for none participation.

### Measurements

Weight was measured by the trained nurses/ interviewers using Seca 761 mechanical scales to the nearest 1kg and height using Leicester Stadiometer to the nearest 0.1 cm. Blood pressure (BP) was measured using the Omron M4-1, for participants who had been resting for at least 15 minutes before measurement, in sitting position using appropriate cuffs. Three measurements of systolic blood pressure, diastolic blood pressure and heart rate were recorded at an interval of 3 minutes. Blood pressure was taken as the mean of the second and third reading. All measurements were performed using calibrated instruments.

### Survey samples

After performing the measurements, phlebotomy was performed by a nurse, using a vacutainer needle with use of a tourniquet where necessary on one of the veins of the arm on the cubital fossae. Blood was drawn from the respondent in the seated position. Five millilitres of blood in an EDTA tube and 5 mls of blood in a plain tube (labelled with a unique identifier linking the sample to an individual patient) were drawn following MRC clinical laboratory protocols. After drawing the blood, blood tubes were inverted 5-6 times to ensure mixing of the clot activator with blood in the plain tubes or mixing of EDTA anticoagulant with blood to prevent clotting in the EDTA tube. Blood samples were drawn between 8.30 am-2.30 pm each day. Two consecutive stool samples (one sample collected by study participant the evening prior to the interview and another in the morning on the day of the interview) were obtained in stool bottles (labelled with a unique identifier) which had been availed to study participants a day prior to the interviews, and instructions on how to fill them made. The samples were stored in cold boxes and transported on the same day to the Kyamulibwa clinic laboratory. In the Kyamulibwa laboratory, plain blood tubes were packed and transported on the same day to the MRC/UVRI Uganda Research Unit on AIDS main laboratory in Entebbe for typing anaemia.

### Laboratory methods

Haemoglobin measurement, malaria tests and stool examinations were performed at the GPC laboratory in Kyamulibwa. . Haemoglobin was measured in the Kyamulibwa laboratory on the same day that the EDTA sample was collected as part of FBC using a Coulter AC.T 5 Diff CP analyser. Anaemia was defined using the WHO criteria: haemoglobin less than130 g/L in males or less than120 g/L in females = no anaemia; haemoglobin ≥110 to <130 g/L in males or ≥110 to <120 g/L in females = mild anaemia; haemoglobin ≥80 to <110 g/L in males or females = moderate anaemia; haemoglobin <80 g/L in males or females = severe anaemia [1]. Thick and thin blood films, Giemsa-stained were prepared from the EDTA blood on the same day the blood sample was collected and examined for malaria parasite and types. Malaria was defined as having a positive blood slide for malaria parasites. Two slides per stool sample were examined for hook worm ova using the Kato Katz method [15]. 

### Anaemia typing

Serum ferritin, serum folate and serum vitamin B 12 were measured using Cobas e411 assay (Roche Diagnostics, Mannheim Germany. Low vitamin B-12 was defined as a concentration < 148 pmol/l [16] and low folate as a concentration of < 3.7nmol/l [17]. C-reactive protein was measured using a standard assay range on the Roche Integra 400 plus analyser (Roche Diagnostics Mannheim Germany). C-reactive protein level greater than 10mg/dl was used as a marker for chronic inflammation in those with iron deficiency anaemia and those with anaemia of chronic disease.

In typing anaemia, we used the cause rather than the microscopic picture of the red blood cells. This was done in order to compare our findings with published studies on anaemia in older people most of which have used the cause rather than the microscopic picture to type anaemia. Since we did not identify any published serum ferritin cut-offs to predict iron deficiency anaemia in older people in Sub Saharan Africa, we used three criteria to determine iron deficiency anaemia: 1) WHO recommendation of serum ferritin levels less than 15 µg/dl in Women and less than 10 µg/dl in Men [18], 2) the criteria that has been used in studies of anaemia in young adults in Africa of serum ferritin levels below 30µ/dl for both Men and Women [19], 3) the criterion that is recommended for older people in high income countries of serum ferritin below 45µg/dl for both Men and Women [20].

 Using different criteria for the determination of iron deficiency anaemia affects the proportion of the different types of anaemia in each category. Since serum ferritin increases with age and infection [21], we finally reported the typing of iron deficiency anaemia and anaemia of chronic disease using the criterion used by studies in high income countries. Although we think this is not the best for older people in Sub Saharan Africa where infections are prevalent, it takes into account the increase of serum ferritin with age.

### HIV testing

HIV testing was done using an algorithm for HIV testing that is recommended by the Uganda Ministry of health. The algorithm for HIV rapid testing consisted of an initial screening with the rapid test Determine HIV1/2. If the test result was negative the participant was given a diagnosis of HIV negative with no further rapid testing. If the test result was positive the sample was retested with the rapid test HIV 1/2 Stat-Pak. If both tests gave a positive result the participant was given a diagnosis of HIV positive with no further rapid testing. If the tests gave discordant results (one positive and the other negative), the sample was further evaluated with the rapid test Uni-Gold Recombinant HIV-1/2. For those samples assessed by all three tests, two positive test results were interpreted as a positive diagnosis. If two of the three tests gave negative results then the participant was diagnosed as being negative for HIV. 

### Quality control

All the laboratory tests were performed by trained laboratory technologists following laid down operating procedures of the MRC/UVRI laboratories and following a laboratory analytical plan which was developed before the commencement of the study. External quality control is carried out on MRC/UVRI Uganda Research Unit on AIDS laboratories by National Health Laboratory Services (South Africa), (12 cycles per year) and Royal College Pathologists Australia for the automated machines (2 cycles per year). In addition to these, laboratory audits are conducted on the laboratories (bi annually) by Contract Laboratory Services South Africa and Qualogy (UK) for good clinical laboratory practice annually.

### Data management and analysis

Data were uploaded from the UMPCs onto a networked computer and automatically backed up onto the server every evening at the Kyamulibwa field station. The following morning, the data were checked by one of the authors (JM) for missing data and erroneous inputs. Data were analysed using STATA 11 (Stata Corp, College Park, TX, USA). 

Baseline characteristics of the study cohort were tabulated by sex. In order to compare our findings with studies conducted in high income countries on anaemia in older people, we calculated the prevalence and 95% confidence interval of anaemia stratified by age group (<65 years and ≥65 years), since most of the studies on anaemia in older people in high income countries have defined older people as those aged 65 years and over [9,22]. We also stratified by sex because most of the studies on anaemia prevalence in older people have shown differences by sex. 

We investigated factors associated with any anaemia (haemoglobin < 130 g/L in males or < 120 g/L in females) using logistic regression to estimate odds ratios (OR) and 95% confidence intervals. Potential determinants of anaemia were examined using a conceptual framework [23] with five levels: sociodemographic factors (age, sex, marital status, educational level and socioeconomic score tertile); smoking and drinking; anthropometric factors (body mass index and blood pressure); dietary factors (skipping meals in past week, days skipped ≥1 meal in past week, meat consumption in past week, fruit consumption in past week and vegetable consumption in past week); and clinical factors (hookworm infection, malaria infection, HIV infection and jiggers). Since there was strong evidence that the association between age and anaemia differed by sex, age, sex and their interaction were considered a priori confounders and included in all models. First, sociodemographic factors that were associated with anaemia at p<0.10 in the age- and sex-adjusted analysis were included in a multivariable model; those remaining associated at p<0.10 were retained in a core sociodemographic model. Smoking and drinking factors were added to this core model one by one. Those that were associated with anaemia at p<0.10, after adjusting for sociodemographic factors, were included in a multivariable model and retained if they remained associated at p<0.10. Associations with anthropometric, dietary and clinical factors were determined in a similar way. This strategy allowed us to assess the effects of variables at each level of the framework, adjusted for more distal variables. A final model was obtained by excluding variables one at a time until all remaining variables were associated at p<0.10. In the final model, interactions were considered between sex and the other covariates. Statistical significance was assessed using likelihood ratio tests. 

## Results

### Description of study participants

A total of 15376 people participated in the GPC census for the 2012/2013 annual survey. Out of these, 2012 (854 men and 1158 women) were aged ≥ 50 years and eligible for the anaemia survey. In total 1455 older people (72.3%) participated in the survey but 6 were excluded from this analysis because of uncertainty over their age. The reasons for non-participation included refusals (10%), not found at home at time of survey (13.7%) and 4% of the respondents were severely ill. Survey participants and non-participants were similar in all characteristics apart from age and socio-economic status (results not shown). 

Data were available for 1449 older people of whom 844 (58.2%) were Women (Table 1). The median age of the study participants was 62 years (IQR 50-94). The majority (88.2%) of the study participants had primary or lower primary education. Current smoking was relatively common among Men (31%) but rare among Women (4.3%), while 373 (61.7%) of the Men and 315 (37.3%) of the Women were current drinkers respectively. The population was relatively slim, with only 6.4% of Men and 21.8% of Women overweight. More than one third of Men and Women had hypertension, and the prevalence of HIV was 8.0% in Men and 5.5% in Women. 

**Table 1:** Description of study participants in survey.

	**Males (N=605)**	**Females (N=844)**
**Age (years)**		
50–59	257 (43%)	344 (40.8%)
60–69	155 (26%)	291 (34.5%)
70–79	138 (23%)	149 (17.7%)
80+	55 (9%)	60 (7.1%)
**Marital status^1^**		
Married	427 (70.7%)	276 (32.7%)
Divorced/separated	115 (19.0%)	218 (25.9%)
Widowed	53 (8.8%)	346 (41.0%)
Single (never married)	9 (1.5%)	3 (0.4%)
**Education level**		
None/incomplete primary	125 (20.7%)	281 (33.3%)
Primary	372 (61.5%)	500 (59.2%)
Secondary	90 (14.9%)	45 (5.3%)
Above secondary	18 (3.0%)	18 (2.1%)
**SES score tertile^2^**		
Low	186 (36.7%)	253 (33.8%)
Middle	175 (34.5%)	294 (39.3%)
High	146 (28.8%)	201 (26.9%)
**Smoking**		
Current smoker	188 (31.1%)	36 (4.3%)
Past smoker	153 (25.3%)	31 (3.7%)
Never smoked	264 (43.6%)	777 (92.0%)
**Chewing tobacco^3^**		
Current user	112 (18.5%)	184 (21.8%)
Past regular user	6 (1.0%)	12 (1.4%)
Never regular user/never used	487 (80.5%)	647 (76.7%)
**Alcohol consumption**		
Current drinker	373 (61.7%)	315 (37.3%)
No drinking in past year	100 (16.5%)	221 (26.2%)
Never drinker	132 (21.8%)	308 (36.5%)
**BMI (kg/m^2^) ^4^**		
Underweight (<18.5)	182 (30.4%)	133 (16.1%)
Normal (18.5–24.9)	379 (63.3%)	515 (62.2%)
Overweight (25.0–29.9)	37 (6.2%)	134 (16.2%)
Obese (≥ 30)	1 (0.2%)	46 (5.6%)
**Blood pressure group**		
Normal	170 (28.1%)	222 (26.3%)
Pre-hypertension	214 (35.4%)	299 (35.4%)
Stage I hypertension	152 (25.1%)	191 (22.6%)
Stage II hypertension	69 (11.4%)	132 (15.6%)
**HIV serostatus^5^**		
Negative	554 (92.0%)	793 (94.5%)
Positive	48 (8.0%)	46 (5.5%)

^1^ Missing marital status for 1 male and 1 female. ^2^ SES score computed from asset index based on household ownership of items in Round 20; missing data for 98 males and 96 females. ^3^ Missing data on chewing tobacco for 1 female. ^4^ Missing data on BMI for 6 males and 16 females. ^5^ Missing data on HIV for 3 males and 5 females.

### Prevalence of anaemia

**Figure 1:**
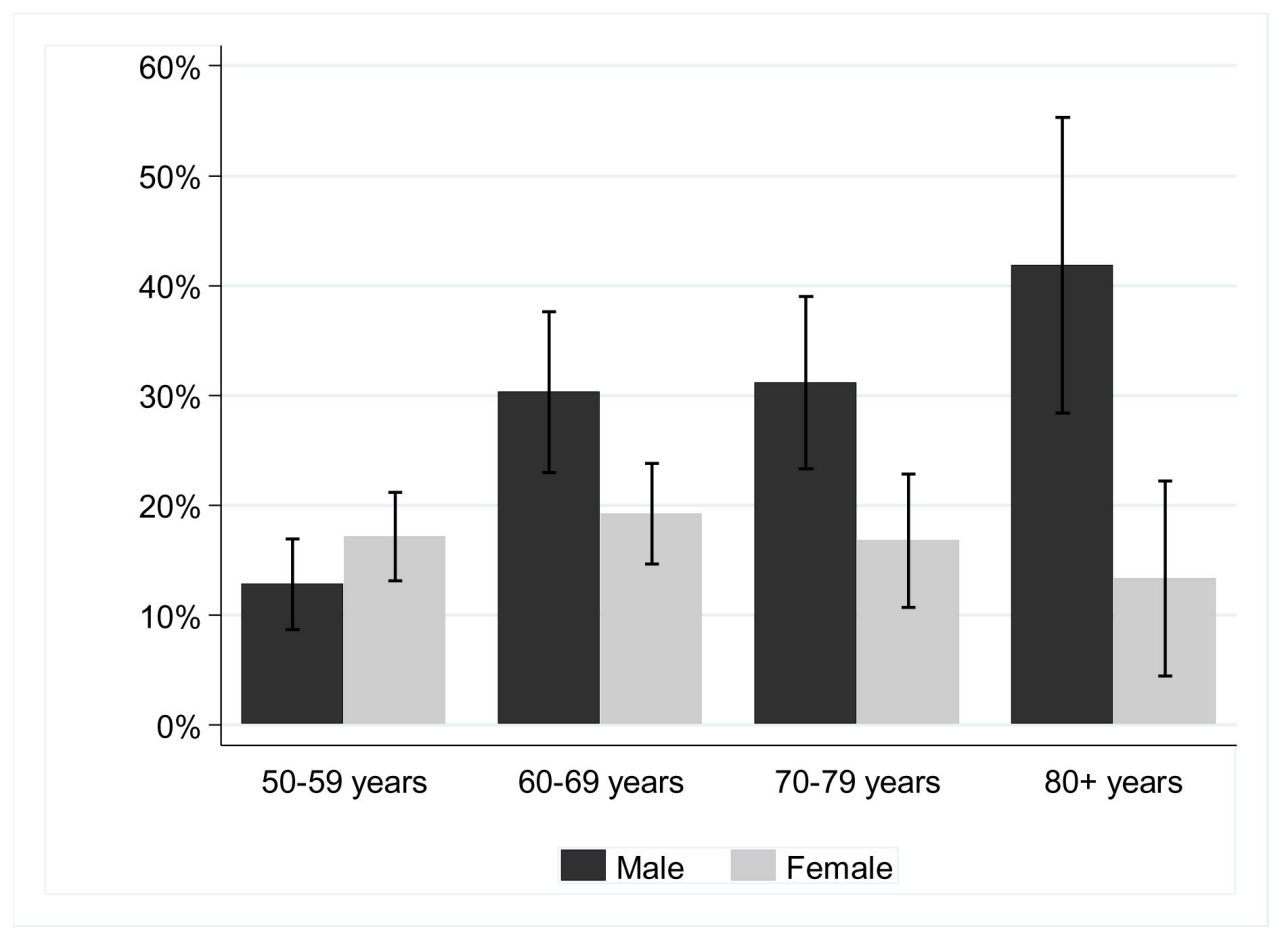
Prevalence of anaemia (and 95% confidence intervals) by age group and sex.

The overall prevalence of anaemia was 20.3% (95% CI 18.2-22.3) and was significantly higher in Men than Women (p=0.002), 24.1% (95% CI=20.7-27.7%) versus 17.5% (95% CI=15.0-20.1%) respectively. When stratified by age <65 and ≥65 years, the prevalence of anaemia in those under 65 years was similar in Men and Women (16.8%, 95% CI=12.9-20.8 and 17.6%, 95% CI=14.3-21.0%, respectively). However, among those ≥65 years, the prevalence of anaemia in Men was nearly double that in those < 65 years, (33.8%, 95% CI=28.1-39.6& and 17.4%, 95% CI=13.4-21.4, respectively). In Men, the prevalence of anaemia increased rapidly with age from the age of 50 (p-trend<0.001, Figure 1), while in Women, there was no evidence of a trend in the prevalence of anaemia with age (p=0.99,). There was strong evidence that the association of age with anaemia differed by sex (p for interaction<0.001).

### Anaemia Types

Among the older people with anaemia, 151 (59.7%) had an explained anaemia or anaemia of unknown cause, 52 (20.6%) had iron deficiency anaemia, 26 (10.3%) had anaemia of chronic disease and 8 (3.2%) had both iron deficiency and vitamin B12 deficiency anaemia. Of those with Iron deficiency anaemia 3 (5.8%) had raised CRP levels compared to 2 (7.7%) of those with anaemia of chronic disease. Other types of anaemia were not common (table 2).

**Table 2:** Types of anaemia among the older people.

	WHO criteria	African studies	Criteria for Older people
Iron deficiency anaemia only (IDA)	8 (3.2%)	35 (13.8%)	52 (20.6%)
Anaemia of chronic disease (ACD)	70 (27.7%)	43 (17.0%)	26 (10.3%)
Folate deficiency anaemia only	8 (3.2%)	7 (2.8%)	7 (2.8%)
Vitamin B12 deficiency anaemia only	13 (5.1%)	11 (4.3%)	8 (3.2%)
IDA and Folate deficiency anaemia	0	1 (0.4%)	1 (0.4%)
IDA and Vitamin B12 deficiency anaemia	3 (1.2%)	5 (2.0%)	8 (3.2%)
Folate and Vitamin B12 deficiency anaemia	0	0	0
Un known cause	151 (59.7%)	151 (59.7%)	151 (59.7%)
**Total**	**N=253^1^**	**N=253^1^**	**N=253^1^**

^1^ Missing results for 40 participants with anaemia

### Factors associated with anaemia among the older people

After adjusting for age and sex (Table 3), the odds of having anaemia were doubled in those who were 80 years and above compared to those aged 50-59 and were lower in Women compared to Men (adj OR 0.66, 95%CI 0.51-0.86). Anaemia was more common in those who were not married compared to the married or in current smokers as compared to those who had never smoked. The odds of anaemia were also higher in those who had drank alcohol in the past year compared to those who had not drank alcohol, and the odds of anaemia were higher in those who had consumed <1 serving of fruit per day . Considering the clinical factors, the odds of having anaemia were also higher in older people with: any hookworm infection (adj OR 1.35, 95% CI 1.03-1.78), heavy hookworm infection (adj OR 3.43, 95%CI 1.77–6.68 comparing heavy infection to no infection), malaria infection (adj OR 3.82, 95%CI 2.02–7.24), HIV infection (adj OR 1.98, 95%CI 1.23-3.18) and those with jiggers (adj OR 2.04, 95%CI 1.04-4.01) compared to those not infected. However, the odds of anaemia were lower among older people with elevated blood pressure than in those with normal blood pressure (adj OR=0.41, CI=0.26-0.66, comparing stage II hypertension to normal BP).

**Table 3:** Association of factors with anaemia.

	**n with anaemia / total participants (%)**		**Age & sex-adjusted OR (95% CI)**
**SOCIO-DEMOGRAPHIC FACTORS**			
**Age (years)**			P<0.001
50–59	92 / 601 (15.3%)		1
60–69	103 / 446 (23.1%)		1.72 [1.26, 2.36]
70–79	68 / 287 (23.7%)		1.69 [1.19, 2.40]
80+	31 / 115 (27.0%)		2.01 [1.26, 3.21]
**Sex**			P=0.002
Male	146 / 605 (24.1%)		1
Female	148 / 844 (17.5%)		0.66 [0.51, 0.86]
**Marital status**			P=0.005
Married	128 / 703 (18.2%)		1
Unmarried	165 / 735 (22.5%)		1.51 [1.13, 2.02]
**Education level**			P=0.66
Secondary or above	30 / 171 (17.5%)		1
Primary	177 / 872 (20.3%)		1.20 [0.78, 1.87]
None/incomplete primary	87 / 406 (21.4%)		1.24 [0.76, 2.01]
**SES score tertile**			P=0.006
High	53 / 347 (15.3%)		1
Middle	97 / 469 (20.7%)		1.39 [0.96, 2.01]
Low	114 / 439 (26.0%)		1.80 [1.25, 2.60]
**SMOKING AND DRINKING**			
**Smoking**			P=0.003
Never smoked	179 / 1041 (17.2%)		1
Past smoker	51 / 184 (27.7%)		1.63 [1.09, 2.45]
Current smoker	64 / 224 (28.6%)		1.83 [1.25, 2.67]
**Chewing tobacco**			P=0.11
Past user/never used	222 / 1152 (19.3%)		1
Current user	72 / 296 (24.3%)		1.29 [0.95, 1.76]
**Alcohol consumption**			P=0.01
Never drinker	66 / 440 (15.0%)		1
No drinking in past year	78 / 321 (24.3%)		1.72 [1.19, 2.49]
Current drinker	150 / 688 (21.8%)		1.45 [1.04, 2.01]
**ANTHROPOMETRY**			
**BMI (kg/m^2^**)			P=0.63
Underweight (<18.5)	72 / 315 (22.9%)		1.07 [0.78,1.46]
Normal (18.5–24.9)	179 / 894 (20.0%)		1
Overweight (25.0–29.9)	30 / 171 (17.5%)		0.98 [0.63,1.51]
Obese (≥ 30)	5 / 47 (10.6%)		0.57 [0.22,1.49]
**Blood pressure group**			P<0.001
Normal	100 / 392 (25.5%)		1
Pre-hypertension	100 / 513 (19.5%)		0.69 [0.50,0.95]
Stage I hypertension	65 / 343 (19.0%)		0.60 [0.42,0.86]
Stage II hypertension	29 / 201 (14.4%)		0.41 [0.26,0.66]
**DIET**			
**Skipped any meals in past week**			P=0.25
No	169 / 873 (19.4%)		1
Yes	125 / 576 (21.7%)		1.17 [0.90, 1.52]
**Days skipped ≥1 meal in past week**			P=0.30
None	169 / 873 (19.4%)		1
1-2	51 / 239 (21.3%)		1.15 [0.81, 1.65]
3-6	28 / 153 (18.3%)		0.93 [0.60, 1.46]
Every day	46 / 183 (25.1%)		1.41 [0.96, 2.05]
**Meat consumption in past week**			P=0.63
1+ serving/day	11 / 71 (15.5%)		1
<1 serving/day	133 / 647 (20.6%)		1.38 [0.70, 2.72]
None	149 / 728 (20.5%)		1.36 [0.69, 2.68]
**Fruit consumption in past week**			P=0.02
1+ serving/day	55 / 370 (14.9%)		1
<1 serving/day	136 / 637 (21.4%)		1.52 [1.07, 2.15]
None	103 / 440 (23.4%)		1.65 [1.14, 2.40]
**Vegetable consumption in past week**			P=0.35
1+ serving/day	48 / 278 (17.3%)		1
<1 serving/day	159 / 763 (20.8%)		1.29 [0.90, 1.84]
None	87 / 407 (21.4%)		1.28 [0.86, 1.90]
**CLINICAL FACTORS**			
**Hookworm infection**			P=0.03
No	182 / 977 (18.6%)		1
Yes	109 / 452 (24.1%)		1.35 [1.03,1.78]
**Intensity of hookworm infection^1^**			P=0.004
Not infected	182 / 977 (18.6%)		1
Light infection	82 / 375 (21.9%)		1.22 [0.91,1.64]
Moderate infection	9 / 39 (23.1%)		1.18 [0.55,2.56]
Heavy infection	18 / 38 (47.4%)		3.43 [1.77,6.68]
**Malaria infection**			P<0.001
No	274 / 1408 (19.5%)		1
Yes	20 / 41 (48.8%)		3.82 [2.02,7.24]
**HIV infection**			P=0.007
No	264 / 1347 (19.6%)		1
Yes	28 / 94 (29.8%)		1.98 [1.23,3.18]
**Jiggers**			P=0.04
No	279 / 1409 (19.8%)		1
Yes	15 / 39 (38.5%)		2.04 [1.04,4.01]

^1^ Based on WHO guidelines. Light ≤2000 EPG; moderate >2000 to 4000 EPG; heavy >4000 EPG.

After adjusting for more distal factors (Table 4), anaemia remained associated with being unmarried, lower socio-economic status, and not having consumed fruits in the last week. The odds of having anaemia remained higher in those with heavy hook worm infection, malaria infection and those with HIV infection compared to those not infected. Anaemia remained inversely associated with hypertension category (or increased blood pressure).

**Table 4:** Final multivariable model of factors independently associated with anaemia.

	**Adjusted OR^1^ (95% CI)**	**Adjusted OR^2^ (95% CI)**
**SOCIODEMOGRAPHIC FACTORS^3^**		
**Marital status**	P=0.002	**P=0.04**
Married	1	**1**
Unmarried	1.59 [1.19, 2.13]	**1.37 [1.01, 1.86]**
**SES score tertile**	P=0.008	
High	1	
Middle	1.37 [0.94, 2.00]	
Low	1.77 [1.22, 2.57]	
**SMOKING, DRINKING AND DIET**		
**Alcohol consumption**	P=0.04	**P=0.06**
Never drinker	1	**1**
No drinking in past year	1.66 [1.12, 2.45]	**1.59 [1.08, 2.34]**
Current drinker	1.26 [0.89, 1.79]	**1.32 [0.94, 1.86]**
**ANTHROPOMETRIC FACTORS**		
**Blood pressure group**	P=0.003	**P=0.007**
Normal	1	**1**
Pre-hypertension	0.68 [0.48, 0.97]	**0.66 [0.48, 0.93]**
Stage I hypertension	0.58 [0.39, 0.87]	**0.63 [0.43, 0.92]**
Stage II hypertension	0.42 [0.25, 0.70]	**0.47 [0.29, 0.77]**
**DIETARY FACTORS**		
**Fruit consumption in past week**	P=0.03	**P=0.04**
1+ serving/day	1	**1**
<1 serving/day	1.66 [1.13, 2.45]	**1.50 [1.05, 2.16]**
None	1.57 [1.04, 2.38]	**1.55 [1.05, 2.29]**
**CLINICAL FACTORS**		
**Hookworm infection**	P=0.04	P=0.08**^3^**
No	1	1
Yes	1.37 [1.02, 1.85]	1.29 [0.97, 1.71]
**Intensity of hookworm infection**	P=0.006	**P=0.008**
Not infected	1	**1**
Light infection	1.25 [0.90, 1.72]	**1.16 [0.85, 1.58]**
Moderate infection	1.08 [0.48, 2.43]	**1.07 [0.47, 2.41]**
Heavy infection	3.75 [1.78, 7.90]	**3.45 [1.73, 6.91]**
**Malaria infection**	P=0.04	**P<0.001**
No	1	**1**
Yes	2.19 [1.05, 4.58]	**3.49 [1.78, 6.82]**
**HIV infection**	P=0.07	**P=0.003**
No	1	**1**
Yes	1.65 [0.97, 2.80]	**2.17 [1.32, 3.57]**

^1^ Adjusted for age group, sex and their interaction, and all variables associated at p<0.10 at the preceding level of the conceptual framework, as shown in the table. ^2^ Adjusted for age group, sex and their interaction, and all independent predictors of anaemia (variables in bold): marital status, alcohol, fruit, blood pressure, hookworm intensity, malaria and HIV infection. ^3^ Adjusted for all variables listed in footnote 2, except intensity of hookworm infection.

In the final model (Table 4), anaemia remained associated with malaria infection (OR 3.49, 95%CI 1.78-6.82), HIV infection (OR 2.17, 95%CI 1.32-3.57) and heavy hookworm infection (OR 3.45, 95%CI 1.73-6.91). Those who had not consumed any fruits in the past week were 1.5 times more likely to have anaemia as compared to those who had one or more servings of fruit in the last week (OR 1.55, 95%CI 1.05-2.29). Those with hypertension stage II were less likely to have anaemia as compared to those with normal blood pressure. (OR 0.47, 95%CI 0.29-0.77). Being unmarried remained associated with anaemia (OR 1.37, 95%CI 1.01-1.86), and there was some evidence that the association between being unmarried and anaemia was stronger in males than in females (p for interaction = 0.01). The association between anaemia with malaria and hookworm was stronger in females but there was no evidence of significant effect modification.

## Discussion

We have established that the prevalence of anaemia in older people in this study population increased with age from 50 years and almost doubled by 65 years of age in men. While the prevalence of anaemia in women increased with age after 50 years, it remained almost constant after 65 years. Contrary to the thinking that most of the anaemia in Uganda is due to iron deficiency [24], anaemia of unknown cause was responsible for more than a half of all the anaemia in older people in this study population. The major predictors of anaemia among the older people were heavy hookworm infection, malaria infection, HIV infection and non-consumption of fruits. 

The increase in the prevalence of anaemia with age and the sex differences in the prevalence of anaemia are consistent with studies conducted in the community in older people in high income countries, including in the USA [9] and Italy [25]. Two studies conducted in the Scandinavian countries among older people aged 70 and above demonstrated that haemoglobin levels decline with ageing, with the declines less pronounced in women than in men [26,27]. The reasons for the decline of haemoglobin concentrations with age and why this decline is more pronounced in men than women are not clear. However, it is thought that reductions in bone marrow erythroid precursors [28,29] and decreased responsiveness of these precursors to stimulatory growth factors with advanced age [30] may be responsible. The explanation that has been previously given for the high prevalence of anaemia in men as compared to women in older people, are the thresholds WHO used to define anaemia as a HB less than 13g/dl in adult men and less than 12g/dl in adult Women [8,9]. Some authors have questioned whether it is logical to still consider women to have a lower Hb than men almost 15 years after menopause [9]. 

The prevalence of anaemia among older people in our study was relatively high compared to estimates from studies conducted in the community in high income countries [22]. Studies conducted in high income countries on anaemia in older people that have included Blacks have also shown high prevalence of anaemia in Blacks as compared to whites [9]. Another possible explanation is the high prevalence of infections in our study setting as compared to high income countries as these are related to the occurrence of anaemia.

There is a big difference between the prevalence of anaemia for men and women (33.8 vs. 17. 4 respectively) in our study for those aged 65 years and above as compared to studies in high income countries. For example in a study that assessed anaemia in older people in the third National Health and Nutrition Examination Survey from 1988-1994, the prevalence of anaemia was 11% for men and 10.2% for women [9]. 

While some studies on anaemia in Uganda have limited the scope of study to iron deficiency anaemia (IDA) [24], this study shows that in addition to IDA, other types of anaemia do exist among older people. Unexplained anaemia was very common in our study and accounted for more than half of all the anaemia among the older people. In high income countries this type of anaemia accounts for 15-45% of the anaemia [9,31-33]. The cause of unexplained anaemia may be attributed to myelodyplastic syndrome [9,34], a disorder that disrupts maturation and differentiation of haematopoietic precursor cells leading to poor quality and quantity of cells in the peripheral blood [35]. It has also been suggested that it may be linked to reduction in the reserve of haematopoietic progenitors, decreased responsiveness of the progenitors to stimulatory growth factors and reduced erythropoietin production occurring during ageing [36,37]. We may have slightly overestimated the proportion of unexplained anaemia in our study population since we did not measure serum creatinine to estimate the proportion of those with anaemia of chronic kidney disease. However, this may have had little effect on our estimate of the proportion of older people with unexplained anaemia since studies conducted in high income countries have shown that anaemia due to chronic kidney disease is responsible for a small proportion (1-8%) of anaemia in older people [9]. In addition, we did not study the genetic causes of anaemia. Sickle cell anaemia which may be one of the causes of anaemia in Uganda is not common in this study population [38], but also given the condition of health services in Uganda, people with sickle cell anaemia are unlikely to survive to the older age.

This finding of a big proportion of unexplained anaemia among the older people will have implications on the management of anaemia among older people in Uganda. Ideally, clinicians should try to investigate and establish the cause of the anaemia before they can treat the anaemia. From the clinical experience of one of the authors of this paper, this is not commonly done in Uganda either due to lack of facilities to fully investigate anaemia at most of the health centers or to the belief that most of the anaemia in Uganda is due to iron deficiency. In these circumstances, most of the anaemia in older people in Uganda is treated as iron deficiency anaemia, especially at lower levels of health care where most of the older people go.

Iron deficiency anaemia was also common in our study population. In high income countries, IDA accounts for between 15-23% of all the anaemia in older people [9,32]. Typing anaemia, especially iron deficiency anaemia and anaemia of chronic disease in older people in Sub Saharan Africa is not easy when using only serum ferritin as an indicator for iron deficiency anaemia or anaemia of chronic disease. Serum ferritin increases with age and infections [21] but we do not have agreed serum ferritin cut offs for predicting iron deficiency anaemia or anaemia of chronic disease in older people in Africa where infectious diseases are prevalent. Where facilities are available, use of other indicators like serum transferrin receptors which are not affected by inflammation is recommended by WHO [18]. The best way to type anaemia of chronic inflammation is to measure serum hepcidin which is an iron-regulatory hormone that is produced in response to inflammatory cytokine IL-6 following inflammation [39]. Assays for measuring serum transferrin receptors and hepcidin are not widely available in Africa, unlike serum ferritin which is available and relatively cheaper compared to the other two. If serum ferritin is to be used in typing anaemia in older people in Sub Saharan Africa, there is need for further studies to be conducted in older people in Sub Saharan Africa to establish the appropriate cut-off levels for serum ferritin that correctly predict iron deficiency anaemia and anaemia of chronic disease in older people. 

Older people who did not consume fruits had an increased risk for anaemia. Fruits especially those that contain vitamin C are good enhancers of iron absorption [40], especially iron obtained from plant sources that may be the common source of iron in our study population but is not bio available iron. Infections like malaria, HIV infection and hookworm infection have previously been pointed out as important causes of iron deficiency anaemia in young adults in low and middle income countries [10]. We did not come across any studies which have previously associated these infections with anaemia in older people. It is therefore important to look for the cause of anaemia in older people where possible so that it can be appropriately managed. Although bleeding tendencies like vomiting blood, blood in stool or urine may cause iron deficiency anaemia, these were not common in our study population. 

Hypertension was inversely associated with anaemia. The mechanism that my explain this may be that people with a high haemoglobin level are likely to have increased blood viscosity and thus increased blood pressure [41,42]. We do not have a clear explanation as to why the risk of anaemia was low in married people. Probably, this could be related to availability of food in older people that are married unlike unmarried ones where food availability could be an issue.

To the best of our knowledge, this is the first study to estimate the prevalence of anaemia among older people in Uganda and among the few epidemiological studies on anaemia in older people in Africa. This study was carried out in a general population with a large sample size. In addition, a blood sample was drawn from all consenting participants, in contrast to previous studies in Africa [43]. We also believe that our response rate of 72.3% was good given that this study population has participated in annual sero-surveys since 1989 and some older people had study fatigue. We were also able to investigate the role of other factors other than iron deficiency in the development of anaemia that have not been given attention previously. On the other hand, we did not use a quantified food frequency questionnaire to measure the habitual dietary intake of older people in this population because of the lengths of the questionnaire, and the fact that most of the older people in this community are illiterate. This could have reduced our ability to find an association between nutritional habits and anaemia. 

In conclusion, this study has established that anaemia is a common condition among older people in this rural population. Unlike in the young adults, most of the anaemia in older people in this population is unexplained anaemia. Since anaemia, even when mild, has been associated with increased morbidity and mortality when it occurs among older people [44] , there is need to include older people in Uganda in anaemia control programmes. Older people should be educated on good sources of bioavailable iron and encouraged to consume fruits which enhance iron absorption. The strong association between anaemia and infectious diseases calls for inclusion of older people in the Uganda ministry of health programme for the control of soil transmitted helminths. In addition, clinicians should endeavor to screen older people who present to them with malaria or HIV for anaemia. 
